# Maturing Autophagosomes are Transported Towards the Cell Periphery

**DOI:** 10.1007/s10571-021-01116-0

**Published:** 2021-06-09

**Authors:** Anna Hilverling, Eva M. Szegö, Elisabeth Dinter, Diana Cozma, Theodora Saridaki, Björn H. Falkenburger

**Affiliations:** 1 Department of Neurology, RWTH Aachen University, Aachen, Germany; 2 Department of Neurology, Technische Universität Dresden, Dresden, Germany; 3 JARA-Institute Molecular Neuroscience and Neuroimaging, Forschungszentrum Jülich GmbH and RWTH Aachen University, Aachen, Germany; 4 Deutsches Zentrum Für Neurodegenerative Erkrankungen, Dresden, Germany

**Keywords:** Autophagy, α-Synuclein, MTOC, Time-lapse microscopy, Autolysosomes, Lysosomes, Amphisomes

## Abstract

Autophagosome maturation comprises fusion with lysosomes and acidification. It is a critical step in the degradation of cytosolic protein aggregates that characterize many neurodegenerative diseases. In order to better understand this process, we studied intracellular trafficking of autophagosomes and aggregates of α-synuclein, which characterize Parkinson’s disease and other synucleinopathies. The autophagosomal marker LC3 and the aggregation prone A53T mutant of α-synuclein were tagged by fluorescent proteins and expressed in HEK293T cells and primary astrocytes. The subcellular distribution and movement of these vesicle populations were analyzed by (time-lapse) microscopy. Fusion with lysosomes was assayed using the lysosomal marker LAMP1; vesicles with neutral and acidic luminal pH were discriminated using the RFP-GFP “tandem-fluorescence” tag. With respect to vesicle pH, we observed that neutral autophagosomes, marked by LC3 or synuclein, were located more frequently in the cell center, and acidic autophagosomes were observed more frequently in the cell periphery. Acidic autophagosomes were transported towards the cell periphery more often, indicating that acidification occurs in the cell center before transport to the periphery. With respect to autolysosomal fusion, we found that lysosomes preferentially moved towards the cell center, whereas autolysosomes moved towards the cell periphery, suggesting a cycle where lysosomes are generated in the periphery and fuse to autophagosomes in the cell center. Unexpectedly, many acidic autophagosomes were negative for LAMP1, indicating that acidification does not require fusion to lysosomes. Moreover, we found both neutral and acidic vesicles positive for LAMP1, consistent with delayed acidification of the autolysosome lumen. Individual steps of aggregate clearance thus occur in dedicated cellular regions. During aggregate clearance, autophagosomes and autolysosomes form in the center and are transported towards the periphery during maturation. In this process, luminal pH could regulate the direction of vesicle transport.

**Graphic Abstract:**

(1) Transport and location of autophagosomes depend on luminal pH: Acidic autophagosomes are preferentially transported to the cell periphery, causing more acidic autophagosomes in the cell periphery and more neutral autophagosomes at the microtubule organizing center (MTOC). (2) Autolysosomes are transported to the cell periphery and lysosomes to the MTOC, suggesting spatial segregation of lysosome reformation and autolysosome fusion. (3) Synuclein aggregates are preferentially located at the MTOC and synuclein-containing vesicles in the cell periphery, consistent with transport of aggregates to the MTOC for autophagy.
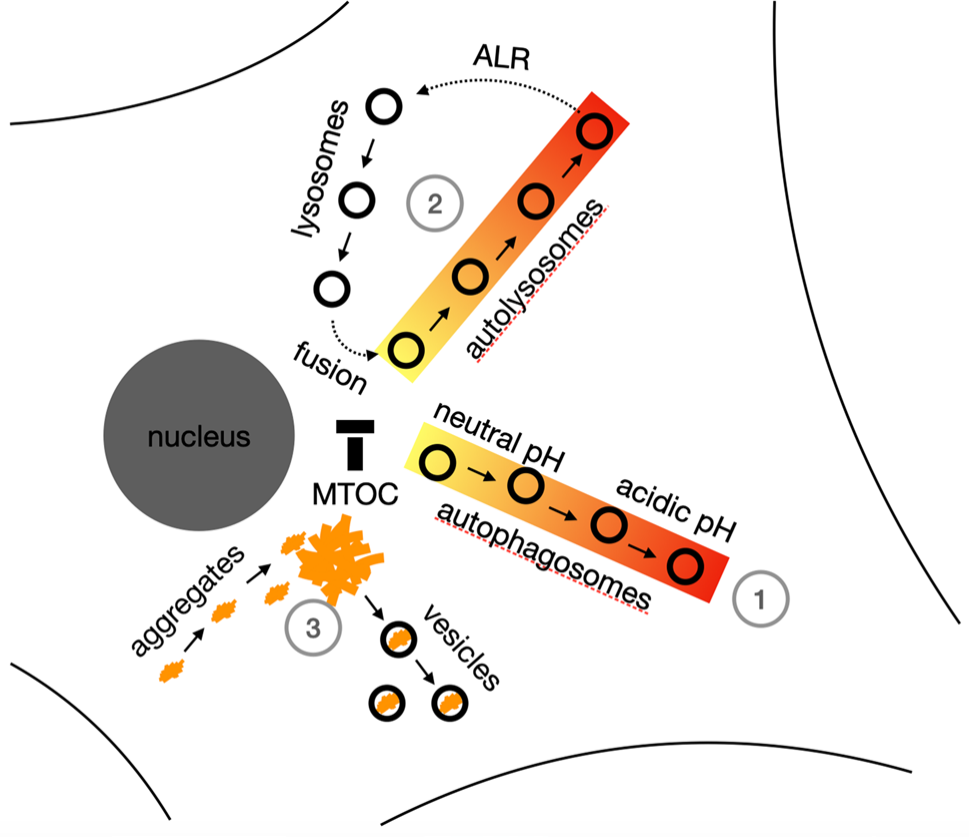

**Supplementary Information:**

The online version contains supplementary material available at 10.1007/s10571-021-01116-0.

## Introduction

Many neurodegenerative diseases are characterized by protein misfolding and the accumulation of protein aggregates, hence the terms “protein misfolding diseases” and “aggregopathies.” Parkinson’s disease (PD), for instance, is characterized by cytoplasmic aggregates of α-synuclein (Spillantini et al. 1997). In most of these diseases, protein aggregates accumulate in neurons. Yet, the cellular mechanisms of protein quality control are highly conserved among eucaryotic cells, and important insight into the pathobiology of protein aggregates has been obtained in yeast (Hill et al. 2017). Accordingly, some protein misfolding diseases primarily affect non-neuronal cells, including multisystem atrophy, which is characterized by α-synuclein aggregates in glia, inclusion body myositis, and senile cardiac amyloidosis. In this work, we focus on cellular mechanisms that mediate the removal of protein aggregates, a process that is relevant for all of these diseases.

Using fluorescently tagged A53T-α-synuclein and time-lapse microscopy, we previously observed that cells are able to remove aggregates of α-synuclein (Opazo et al. 2008; Dinter et al. 2016; Saridaki et al. 2018). Aggregates are transported towards the perinuclear microtubule organizing center (MTOC) before degradation (Opazo et al. 2008; Saridaki et al. 2018), consistent with the previous findings by others that dynein motors mediate transport of misfolded green fluorescent protein (Johnston et al. 2002), and that mutations or blockade of the retrograde motor dynein impair clearance of A53T-α-synuclein (Ravikumar et al. 2005). The perinuclear site that coordinates transport and clearance of aggregates is called aggresome in mammalian cells (Kopito 2000) and juxtanuclear quality control site (JUNQ) in yeast (Kaganovich et al. 2008; Hill et al. 2017). Yeast cells show a second site of aggregate accumulation with distinct properties that is called insoluble protein deposit (IPOD) and bears similarities to p62 bodies in mammalian cells (Johansen and Lamark 2011).

Clearance of α-synuclein aggregates occurs by (macro) autophagy (Webb et al. 2003; Ebrahimi-Fakhari et al. 2011; Watanabe et al. 2012) where damaged proteins and dysfunctional organelles are engulfed by precursor membranes, forming double-membrane autophagosomes that subsequently fuse with lysosomes to degrade their content (Johansen and Lamark 2011). In this context, the function of accumulating protein aggregates at the MTOC is to make autophagy more efficient by bringing together substrates and precursor membranes (Iwata et al. 2005).

The use of fluorescence microscopy thus allowed us to resolve spatial aspects of aggregate clearance. Because cells use spatial segregation to facilitate distinct metabolic steps, resolving spatial aspects can be helpful to unravel cell biological mechanisms. Spatial aspects are particularly interesting in the context of neurodegenerative diseases because the compartmentalization into a vast network of neuronal arborizations could be one of the reasons why neurons are particularly vulnerable to the accumulation and toxicity of protein aggregates. In resting cells, lysosomes are mainly found in the periphery, but have also been described at the perinuclear MTOC (Matteoni and Kreis 1987; Korolchuk et al. 2011). In starvation-induced autophagy, autophagosomes form randomly in the cytosol and move towards the MTOC where they “meet” lysosomes to form autolysosomes (Jahreiss et al. 2008; Korolchuk et al. 2011; Starling et al. 2016). It has remained unclear, however, to what extent the trafficking events observed for starvation-induced autophagy are applicable to the degradation of aggregates in non-starved cells given that starvation-induced autophagy and specific autophagy of misfolded or ubiquitinated proteins differ in various aspects (Lamark and Johansen 2012; Galluzzi et al. 2017).

In order to better understand the cell biological process of aggregate clearance, we systematically studied the transport and fusion events during clearance of α-synuclein aggregates. We expressed fluorescently tagged combinations of A53T-α-synuclein, the autophagosome marker LC3, and the lysosome marker LAMP1 in HEK293T cells and primary astrocytes. We analyzed their subcellular distribution and studied their intracellular movement by time-lapse microscopy. Vesicles with neutral and acidic luminal pH were discriminated using the RFP-GFP “tandem-fluorescence” tag (Pankiv et al. 2007; Kimura et al. 2007).

## Methods

### DNA Constructs

A53T-α-synuclein was flexibly tagged by either mCherry, CFP, RFP-GFP tandem-fluorescence (TFL), or mCherry-FRB using the interaction of a six amino acid PDZ-binding motif, added to the C-terminus of synuclein, with the corresponding PDZ domain fused to mCherry or TFL. Flexible tagging of α-synuclein was achieved by the interaction of six amino acids added to its C-terminus with a coexpressed PDZ domain fused to TFL. The tagging strategy was used previously and allows visualization of α-synuclein-positive puncta in fixed cells and their tracking in life cells (Opazo et al. 2008; Karpinar et al. 2009; Krumova et al. 2011; Dinter et al. 2016; Saridaki et al. 2018).

To follow puncta decorated by LC3 (Microtubule-associated protein 1A/1B-light chain 3), we used ptfl-LC3 (CMV-mRFP1-EGFP-LC3, a generous gift from Tamotsu Yoshimori, obtained through Addgene, #21074). For LC3 tagged by red fluorescence, we created pcDNA3.1-mCherry-LC3 by cloning mCherry into pcDNA3.1-LC3 using NotI and XbaI. To follow puncta decorated by LAMP1 (Lysosomal-associated membrane protein 1), pEGFP-N1-LAMP1 was generated from pmRFP-N1-LAMP1 (generous gift from Walther Mothes, obtained through Addgene, #1817) by replacing pRFP with EGFP using BglII and AgeI. For recruitment of GFP to α-synuclein tagged by mCherry-FRB by chemically induced dimerization, we used EGFP-FKBP from Takanari Inoue (Johns Hopkins University, Baltimore, USA).

### Cell Culture

HEK293T cells were cultured in DMEM (Dulbecco’s modified Eagle´s medium; P04-03550, PAN-Biotech, Aidenbach, Germany) with 10% FCS (fetal calf serum). Cells were transiently transfected using Metafectene (Biontex Laboratories, Martinsried, Germany) following the manufacturer's instructions. The cultures were maintained at 37 °C in an incubator with 5% CO_2_ and 30% humidity. The cell line was validated in November 2017 by analysis of 21 genetic loci (Promega, PowerPlex 21 PCR Kit carried out by Eurofins Medigenomix Forensik, Ebersberg, Germany).

HEK293T cells were fixed 24 h after transfection: Cells were washed three times in 20 °C PBS (phosphate buffered saline; PAN-Biotech, Aidenbach, Germany) and incubated in a solution containing 4% paraformaldehyde (Merck KGaA, Darmstadt, Germany) and 5% Sucrose (Sigma-Aldrich Chemie GmbH, Taufkirchen, Germany) in PBS for 10 min. Coverslips were washed three times with 4 °C PBS, once with sterile water (ddH_2_O) and dried for 1 h. Coverslips were mounted using Fluoromount-G (Southern Biotech, Birmingham, USA). Images in the green and red fluorescence channels were acquired by an Olympus IX81 microscope (60 × oil objective, NA 1.3).

Primary astrocyte cultures were prepared from transgenic mice expressing LC3-TFL from a CAG promoter (C57BL/6-Tg(CAG-RFP/EGFP/Map1lc3b)1Hill/J, Stock 027139, Jackson Laboratory, Bar Harbor, USA). Cultures were obtained from P1–P3 (mixed sex) mice as previously described (Fedoroff and Richardson 2001). Briefly, after removing the meninges, cerebral cortices were mechanically dissected and dissociated by incubation with 0.05% Trypsin–EDTA (ThermoFisher Scientific, Waltham, USA) for 15 min at 37 °C. Digestion was terminated with FCS and cell suspensions were centrifuged (15 g, 5 min). Cell pellets were titurated in DMEM with 10% FCS, and plated in T-75 flasks (Falcon) coated with poly-L-ornithine (100 µg/ml, Sigma). Astrocytes were first cultured for 5–7 days. Microglia growing on the top of the astrocyte layer were mechanically detached (shaking with 250 rpm for 60 min) from the confluent cultures. After replating in a T-75 flask, astrocytes were expanded for additional 7–10 days. For imaging, 25,000 cells/well (24-well plate) were seeded on poly-l-ornithine-coated glass coverslips, and cultured for further 2–3 days before fixation.

To determine the distribution of α-synuclein-positive vesicles, astrocytes were transiently transfected with CFP-tagged α-synuclein using JetOptimus DNA Transfection Reagent (Polyplus, Illkirch, France), according to the manufacturer’s protocol (250 ng DNA per well). Transfected astrocytes were fixed 48 h after transfection.

### Immunocytochemistry

For immunocytochemistry, HEK293T cells were grown on poly-l-Lysin-coated glass coverslips in 24-well plastic plates. Cells were fixed 24 h after transfection (see above). Coverslips were washed two times in 4 °C PBS and once in 0.1% Triton X-100 (Carl Roth GmbH, Karlsruhe, Germany) in PBS. Coverslips were subsequently incubated for 30 min in blocking solution: 1% BSA (Albumin Fraction V, Carl Roth GmbH, Karlsruhe, Germany) and 0.1% Triton X-100 in PBS. The primary antibody for LAMP1 (Abcam ab24170) was diluted 1:150 in 0.2% BSA (in PBS) and incubated overnight at 4 °C in a humid chamber. On the next day, coverslips were washed three times with 0.2% BSA (in PBS). The Cy7-conjugated secondary antibody (Genecopoeia L144A, 1:100 in 0.2% BSA) was incubated for 4 h at 20 °C in a humid chamber. After washing once in 0.2% BSA (in PBS), once in PBS, and once in ddH_2_O coverslips were mounted using Fluoromount-G. Images in the green, red, and Cy7 channels were acquired by an Olympus IX81 microscope (60 × oil objective, NA 1.3).

Primary astrocytes were incubated for 60 min in blocking solution (1% BSA and 0.1% Triton X-100 in PBS). The primary LAMP1 antibody was used at 1:500 and incubated in 1% BSA (overnight, 4 °C). The Alexa 647-conjugated secondary antibody (Invitrogen, 1:1000 in 1% BSA) as applied for 1 h at 20 °C. After washing in PBS, coverslips were mounted using Fluoromount-G. Images were acquired on a Zeiss Spinning Disc microscope (AxioObserver.Z1, 40 × objective NA 0.95 with Yokogawa CSU-X1M 5000 camera).

### Analysis of Fixed Cells

To determine the subcellular position of puncta, we used two types of analysis. In the first analysis, the position of each particle relative to the nucleus was determined using ImageJ64 (NIH) with the “cell counter” plugin by Kurt De Vos (University of Sheffield, UK). Abundances and distances were subsequently summarized using a custom written macro in IGOR Pro 6 (Wavemetrics, Portland, USA) followed by visualization and statistical analysis in Graph Pad Prism 5 and 7 (GraphPad Software, San Diego, Ca, USA).

**Fig. 1:**
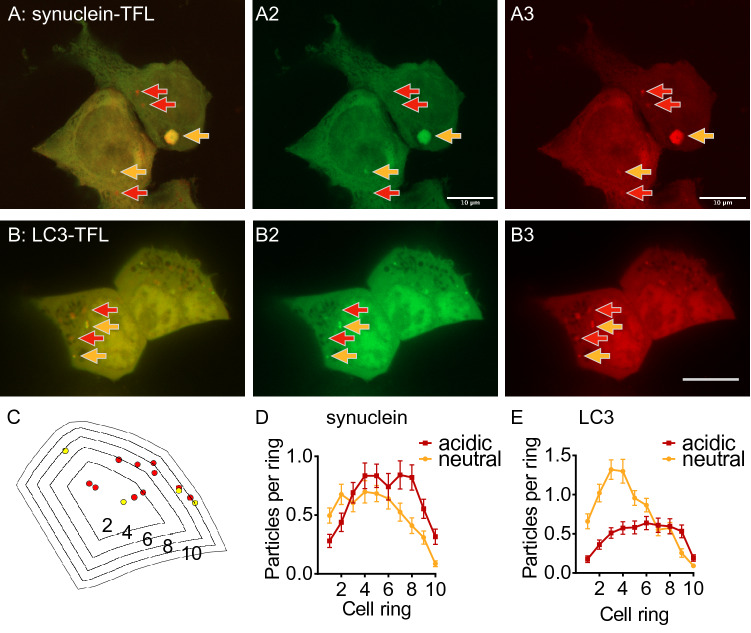
Localization of TFL-tagged α-synuclein and LC3. **A** Image of fixed HEK293T cells expressing A53T-α-synuclein tagged by mRFP-EGFP tandem-fluorescence (TFL). Yellow arrows mark the location of neutral synuclein puncta, i.e., puncta visible in both channels. These puncta appear yellow in the merged image. Red arrows mark the location of acidic puncta, i.e., puncta visible in the red channel but not in the green channel. These puncta appear red in the merged image. Scale bar 10 µm. **B** Image of HEK293T cells expressing LC3-TFL to label autophagosomes. Arrows and scale bar as in (**A**). **C** Outline of the right cell in (**B**) repeatedly eroded to 80, 60, 40, 20% of its initial size, yielding five cell “rings” labeled by 2, 4, 6, 8,10 with 2 being the most central and 10 being the most peripheral ring. Five rings resulting from consecutive erosion by 20% are shown for clarity; analyses were carried out with erosion by 10% yielding 10 rings. **D** Average number of neutral (yellow) and acidic (red) puncta in each ring of *n* = 139 cells from 6 independent experiments with cells transfected as in (**A**). Two-way ANOVA (factors pH and cell ring) showed significant interaction (*p* = 0.001), differences between rings (*p* < 0.0001), and between neutral and acidic puncta (*p* = 0.0012). **E** Shrink analysis for *n* = 141 cells from 6 independent experiments, transfected with LC3-TFL as in (**B**). Two-way ANOVA showed significant interaction (*p* < 0.0001), differences between rings (*p* < 0.0001), and between neutral and acidic puncta (*p* < 0.0001)

A second type of analysis determined the position of puncta in “shrinking” cell outlines (see Fig. 1C) based on two previous publications (Johnson et al. 2016; Starling et al. 2016). The cell was outlined manually in ImageJ as a freehand region of interest (ROI). Using a custom written macro, which is available in the supplement, the cell outline was converted to a binary mask and eroded until the area was 90% of the original area. The outline of this mask was then saved as a second ROI. The mask was further eroded and the outlines corresponding to 80%, 70%, etc. saved. For each ROI, the number of puncta within the ROI was determined, generating a cumulative histogram of the particle distribution with 100% corresponding to the entire cell and 10% corresponding to the most central area. From this cumulative histogram, we then determined the number of puncta for each ring: The first ring was the 10% ROI, the second ring was the difference between to 10% and the 20% ROI, etc.

### Time-Lapse Microscopy

For time-lapse fluorescence microscopy, cells were grown on 24-glass well plates coated by poly-l-lysine and used 18–20 h after transfection. Time-lapse images were acquired with an Olympus IX81 microscope (Olympus, Hamburg, Germany) equipped with an incubator (37 °C, 5% CO_2_, 30% humidity), a 60 × oil objective (NA 1.3), a Hamamatsu CCD Camera (C8484-03G02), and xCellence Software (Version 2.0, Olympus). Images in the green and red channels were acquired twice per second for 2:30 min (300 frames) with an exposure of 100 ms for each channel.

For tracking puncta, we used ImageJ64 with the “manual tracking” plugin by Fabrice Cordelieres (Institute Curie, Orsay, France). The center of the nucleus was recorded with the “cell counter” plugin. The tracking data were then analyzed using a custom written macro in IGOR. The difference between the distance to the nucleus at the beginning of tracking and the distance to the nucleus at the end of tracking was called “convergence.” Puncta with a convergence of less than 0.5 µm were considered immobile. Positive convergence means that puncta moved towards the nucleus and negative convergence means that puncta moved towards the cell periphery.

The longest stretch of directional transport was determined by calculating the distance in µm between all pairs of locations recorded during tracking. For this stretch of transport, we then determined the duration in s and the “net velocity” as stretch/duration.

### Statistical Analysis and Data Visualization

For the quantification of particle distribution in fixed cells, three coverslips were prepared per condition in each experiment and five to seven pictures of each coverslip acquired. Each experiment was carried out at least three times. The total number of cells and the number of independent experiments are noted in the figure legend. For the quantification of time-lapse data, one to three wells of a 24-well glass plate were transfected per condition in each experiment and five to ten movies acquired per well. The number of puncta, cells, and independent experiments are noted in the figure legends. Analysis were carried out with GraphPad Prism. Bars or lines represent mean ± SEM. *p* < 0.05 was considered statistically significant.

## Results

### Subcellular Location Differs Between Neutral and Acidic α-Synuclein Puncta

In order to study location and transport of α-synuclein in different phases of degradation, we expressed in HEK293T cells the pathogenic A53T mutant of α-synuclein flexibly tagged by GFP-RFP tandem-fluorescence (TFL). The TFL tag reports α-synuclein located in an acidic environment by disappearance of the green fluorescence, because GFP is quenched in acidic pH, whereas RFP is more pH resistant. Cells transfected with TFL-tagged A53T-α-synuclein showed fluorescent puncta in both color channels next to diffuse fluorescence in the cytosol (Fig. 1A). Puncta visible only in the red channel (red arrows) represent α-synuclein inside acidic vesicles. Puncta visible in both channels (yellow arrows) can represent α-synuclein in vesicles of neutral pH or cytosolic α-synuclein aggregates, including the perinuclear aggresome (Fig. 1A, right yellow arrow).

In the cell periphery, acidic α-synuclein puncta appeared more common than neutral puncta. In order to quantify this impression, we computed the distribution of puncta across regions of interest (ROI) generated by progressively eroding the cell outline (Fig. 1C). We refer to this analysis as “shrink analysis.” The results are displayed as the average number of puncta per cell ring (Fig. 1D). For neutral α-synuclein puncta, the fraction in the inner 10% of the cell was larger than for acidic puncta, and neutral puncta were more commonly observed in the cell center. Acidic puncta, in contrast, were more commonly observed in the cell periphery (Fig. 1D). There was no significant difference in the number of neutral and acidic puncta per cell. Similar findings were obtained when we compared the distance in µm of each punctum from the center of the nucleus (not shown), but we consider the shrink analysis to better account for the polygonal shape of adherent cells.

α-Synuclein aggregates are cleared by autophagy (Webb et al. 2003; Ebrahimi-Fakhari et al. 2011; Watanabe et al. 2012). We therefore assume that the acidic vesicles containing α-synuclein are autophagosomes. Consistently, we previously observed α-synuclein in vesicles decorated by the late endosome/autophagosome markers Rab7 and FYCO1 (Dinter et al. 2016; Saridaki et al. 2018). In order to determine whether our observations for α-synuclein apply to autophagosomes in general, we expressed the autophagosomal marker LC3 tagged with TFL (Fig. 1B). LC3 is used widely to locate and follow autophagosomes (Kabeya 2000; Jahreiss et al. 2008; Klionsky et al. 2016). Fluorescently tagged LC3 forms visible puncta only when it accumulates on autophagosomal membranes. LC3-positive puncta are therefore considered autophagic vesicles. Using the same analysis (Fig. 1C), neutral LC3 vesicles were more commonly observed in the cell center and acidic LC3 vesicles were more commonly found in the cell periphery (Fig. 1E). Again, similar findings were obtained when analyzing the distance from the nucleus (not shown).

### Acidic Puncta of α-Synuclein Move Towards the Periphery

**Fig. 2:**
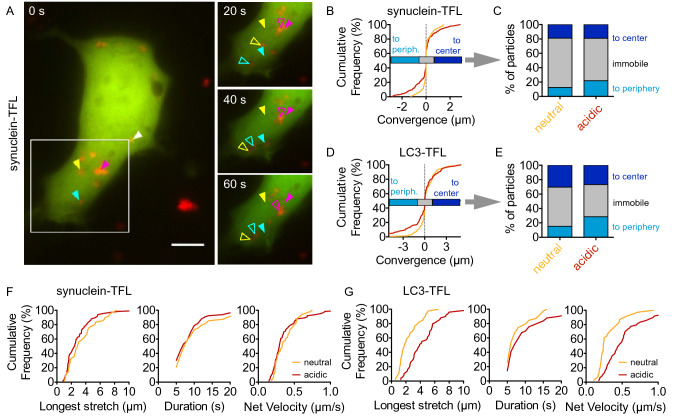
Transport of TFL-tagged α-synuclein and LC3. **A** Sequential images of a living HEK293T cell expressing TFL-tagged A53T-α-synuclein, acquired at the beginning of recording (0 s), and 20, 40, 60 s later, red and green channels merged. White, yellow, and blue arrowheads depict small and large acidic α-synuclein puncta, i.e., puncta that appear red in the merge image. Pink arrowheads depict an α-synuclein cluster, which did not move much. Full arrowheads depict the start position; empty arrowheads depict the current particle position at the later time points. Scale bar 10 µm. **B** Cumulative histogram of convergence values for neutral (yellow) and acidic (red) puncta in cells as in (**A**), computed as the difference in the distance from the nucleus between the beginning and the end of tracking. *n* = 170 neutral and *n* = 240 acidic puncta from 65 cells and 3 independent experiments. **C** Data from (**B**) expressed as the percentages of neutral and acidic α-synuclein puncta with movement towards the cell center (convergence > 0.5 µm), towards the cell periphery (convergence <  − 0.5 µm), or immobile (− 0.5 µm < convergence < 0.5 µm). Comparisons by *χ*^2^ test. *p* = 0.018 for “to periphery” vs “not to periphery” and neutral vs. acidic. **D** Cumulative histogram of convergence values computed as for (**B**) from cells transfected with TFL-tagged LC3. *n* = 146 neutral and *n* = 112 acidic vesicles from 39 cells and 3 independent experiments. (**E**) Percentages of neutral and acidic vesicles in the categories as defined for (**C**) with data from (**E**). Comparisons by *χ*^2^ test. *p* = 0.0016 for “immobile” vs. “not immobile” and α-synuclein vs LC3. *p* = 0.026 for “to periphery” vs “not to periphery” and neutral vs. acidic. **F**, **G** Cumulative histograms of the longest stretch of directional transport observed for each particle during tracking (left), the duration of this transport (center), and the speed calculated as stretch/duration (right). Experiments with TFL-tagged A53T-α-synuclein (**F**) and TFL-tagged LC3 (**G**)

There are two possible explanations for the preferential location of acidic vesicles in the cell periphery. Either vesicles acidify in the cell periphery, or acidic vesicles are preferentially transported towards the cell periphery. In order to discriminate between these two possibilities, we carried out time-lapse microscopy in individual living cells expressing TFL-tagged A53T-α-synuclein or TFL-tagged LC3 (Fig. 2). In Fig. 2A, filled arrowheads mark the position of puncta at the beginning of the recording (0 s) and open arrowheads mark the position at later time points (20 s, 40 s, 60 s). At the beginning and at the end of tracking, we determined the distance from the center of the nucleus. The difference between these two values was referred to as “convergence” (Fig. 2B). We defined puncta with a convergence more negative than − 0.5 µm as moving “to the periphery,” puncta with a convergence between − 0.5 µm and + 0.5 µm as “immobile,” and puncta with a convergence more positive than + 0.5 µm as moving “to the center” (Fig. 2C). With TFL-tagged α-synuclein, 60–70% of puncta did not move during the 2.5 min recording (Fig. 2C). Movement towards the cell periphery was more common for acidic α-synuclein puncta than for neutral puncta; movement towards the cell center was not significantly different between neutral and acidic puncta (Fig. 2C). Most α-synuclein particles moved between 2 and 6 µm within 5–10 s (Fig. 2F). The “net velocity” calculated from this ratio is considerably slower than determined for individual events of unidirectional transport in axons. There were no significant differences between neutral and acidic particles (Mann–Whitney test).

We then analyzed the mobility of TFL-tagged LC3 vesicles in the same way (Fig. 2D, E). LC3 vesicles were more mobile than α-synuclein puncta: A higher fraction of LC3 vesicles was mobile (*p* = 0.0016, chi-square test). Acidic LC3 vesicles were particularly mobile. Neutral LC3 vesicles moved a similar distance as α-synuclein particles did (Fig. 2G), but acidic LC3 vesicles moved significantly longer distances during the same time (Mann–Whitney test), resulting in higher “net velocity” calculated from this ratio. Acidic LC3 vesicles moved towards the periphery more often than neutral LC3 vesicles did (Fig. 2E); movement towards the cell center was not significantly different. Taken together, these findings indicate that vesicles already acidify in the cell center, and then move towards the cell periphery.

### Autolysosomes are Found Mainly in the Cell Center

**Fig. 3:**
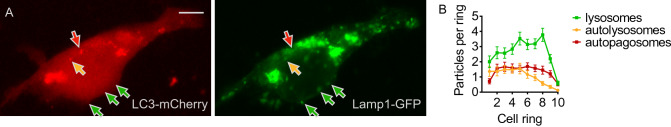
Localization of vesicles tagged by LAMP1 and LC3. **A** Images of fixed HEK293T cells expressing mCherry-tagged autophagosomal marker LC3 and EGFP-tagged lysosomal marker LAMP1. Red arrows indicate the position of autophagosomes, i.e., puncta visible in the red channel but not in the green channel. Green arrows indicate the position of lysosomes, i.e., puncta visible only in the green channel. Yellow arrows indicate autolysosomes, i.e., puncta visible in both channels. Scale bar 10 µm. **B** Average number of puncta per cell ring, using the “shrink analysis” depicted in Fig. 1C, from *n* = 50 cells as in (**A**) and 3 independent experiments. Two-way ANOVA (factors type and cell fraction) showed significant interaction (*p* = 0.0002), differences between rings (*p* < 0.0001), and between neutral and acidic puncta (*p* < 0.0001)

In the experiments presented so far, we assumed that neutral vesicles correspond to autophagosomes and acidic vesicles correspond to autolysosomes as previously reported (Pankiv et al. 2007; Kimura et al. 2007). In order to validate the above findings, we used the autophagosome marker mCherry-LC3 with LAMP1-GFP, a common marker for lysosomal compartments (Shen and Mizushima 2014; Xu and Ren 2015). Using the shrink analysis, we then compared the positions of vesicles only positive for LAMP1-GFP (lysosomes, green arrows in Fig. 3A), only for mCherry-LC3 (autophagosomes, red arrows in Fig. 3A), and double-labeled “overlay” vesicles (autolysosomes, yellow arrows in Fig. 3A). With this method, we observed more lysosomes than either autophagosomes or autolysosomes (Fig. 3B). Autolysosomes were on average located further in the cell center than autophagosomes and lysosomes (Fig. 3B). This finding was unexpected given the results in Fig. 1E. The discrepancy could result from overexpression of LAMP1-GFP in Fig. 3 or by the fact that autophagosomes do not change their pH directly after fusion with LAMP1-positive lysosomes.

### Not All Autolysosomes are Acidic and Vice Versa

**Fig. 4:**
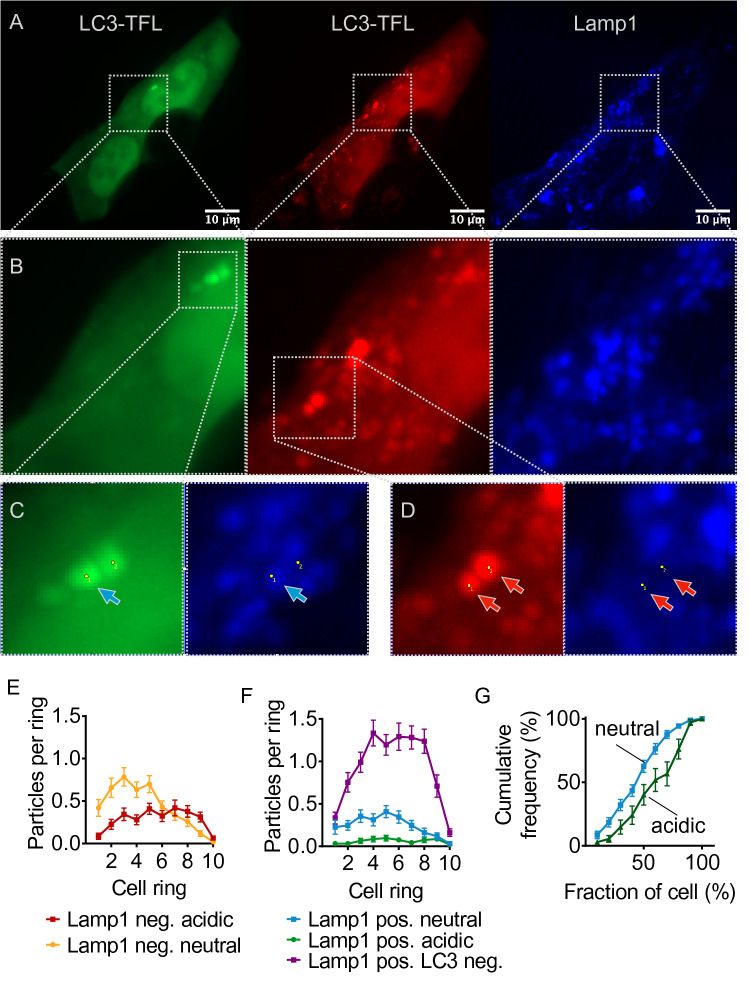
Acidic LC3-tagged vesicles and LAMP1-tagged vesicles are diverse. **A** Image of fixed HEK293T cells expressing TFL-tagged autolysosomal marker LC3 (red and green channels) and stained against LAMP1 (blue channel). Scale bar 10 µm. **B** Inset indicated in (**A**). **C** Inset indicated in **B**. The two dots mark the same positions in both channels. The blue arrow points to an early autolysosome, a punctum that is visible in the green channel (indicating neutral pH) and also visible as a punctum in the blue channel (i.e., positive for LAMP1). The second dot marks a green punctum that is LAMP1 negative because there is no discernable punctum at the same position in the blue channel. **D** Inset indicated in **B**, showing two amphisomes, i.e., autophagosomal vesicles with acidic luminal pH as indicated by the preserved red fluorescence, quenched green fluorescence (visible in B), and LAMP1 negative (no puncta in the blue channel). **E** Average number of neutral (yellow) and acidic (red) puncta negative for LAMP1 per cell ring from *n* = 93 cells as in (A) and 4 independent experiments. **F** Average number of neutral (blue) and acidic (green) puncta positive for LAMP1 from the same cells as in (**C**). Lysosomes (LAMP1 positive, but no green or red fluorescence) depicted in purple. Two-way ANOVA of the combined data from (**D**) and (**E**) (factors type and cell fraction) showed significant interaction (*p* < 0.0001), differences between rings (*p* < 0.0001), and between types of puncta (*p* < 0.0001). **G** Data from (**E**) displayed as cumulative histogram. A higher fraction of neutral LAMP1-positive puncta was found in the more central fraction of the cell (significant at fractions 40–70%)

In order to resolve this discrepancy between the distribution of autolysosomes as defined by acidic vesicles (found in the periphery, Fig. 1E) and autolysosomes as defined by LC3/LAMP1 overlay vesicles (found in the cell center, Fig. 3B), we stained cells expressing TFL-tagged LC3 with an antibody against LAMP1 (Fig. 4A). With this approach we observed vesicles with all possible combinations of labels: We observed neutral LC3 vesicles that were LAMP1 negative as expected for autophagosomes, but some neutral vesicles were LAMP1 positive (Fig. 4C, blue arrow). Similarly, we observed acidic LC3 vesicles that were LAMP1 negative (Fig. 4D, red arrows) in addition to acidic LC3 vesicles that were LAMP1 positive.

We then plotted for LAMP1-negative vesicles (Fig. 4E) and for LAMP1-positive vesicles (Fig. 4F) the location of neutral and acidic vesicles using the shrink analysis. For both populations, neutral vesicles were located further in the cell center than acidic vesicles. For LAMP1-positive vesicles, the difference was better visible using the cumulative histogram (Fig. 4G) than with the individual rings (Fig. 4F). This finding indicates that luminal pH might regulate transport and subcellular location of autophagosomes and autolysosomes. Lysosomes, i.e., vesicles positive for LAMP1 and negative for LC3, were the most abundant vesicle species (purple in Fig. 4F).

The discrepancy between peripheral acidic LC3 vesicles (Fig. 1) and central autolysosomes as defined by the LC3/LAMP1 overlay (Fig. 3) can therefore be resolved by two observations: (1) The majority of LC3/LAMP1 overlay vesicles is in fact neutral (blue > green in Fig. 4F) and preferentially located in the cell center. Neutral LAMP1-positive vesicles likely represent autophagosomes early after fusion with lysosomes, when the internal membrane of the autolysosome is still intact, as recently described by others (Tsuboyama et al. 2016). We therefore refer to this population as early autolysosomes and use mature autolysosomes for acidic LAMP1-positive vesicles. (2) A substantial fraction of LAMP1-negative LC3 vesicles is in fact acidic (Fig. 4E) and located preferentially in the cell periphery. Acidic LAMP1-negative vesicles could represent amphisomes, which are generated by fusion of autophagosomes with endosomes and for which acidification has been described (Sanchez-Wandelmer and Reggiori 2013; Klionsky et al. 2016). We will refer to them as amphisomes from here on but acknowledge that their identity will need to be confirmed in future studies.

**Fig. 5:**
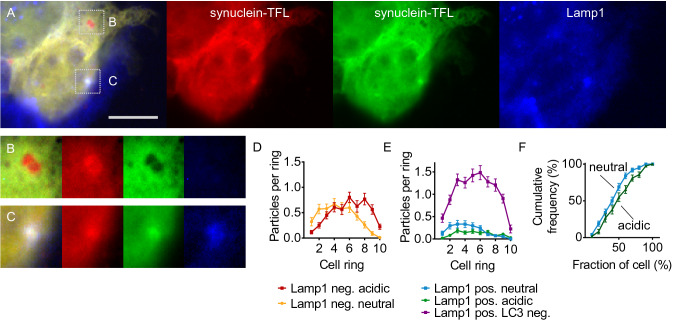
Acidic α-synuclein puncta and LAMP1-tagged vesicles are diverse. **A** Image of fixed HEK293T cells expressing TFL-tagged α-synuclein (red and green channels) and stained against LAMP1 (blue channel). Scale bar 10 µm. **B** Inset indicated in (**A**), showing two amphisomes, i.e., autophagosomal vesicles with acidic luminal pH as indicated by the preserved red and quenched green fluorescence, but negative for LAMP1 (no punctum in blue channel). **C** Inset indicated in (**A**) showing an early autolysosome as indicated by the neutral pH (preserved green fluorescence), but positive for LAMP1. **D** Average number of neutral (yellow) and acidic (red) puncta negative for LAMP1 per cell ring from *n* = 112 cells as in (**A**) and 4 independent experiments. **E** Average number of neutral (blue) and acidic (green) puncta positive for LAMP1 from the same cells as in (**C**). Lysosomes (LAMP1 positive, but no green or red fluorescence) depicted in purple. Two-way ANOVA of the combined data from (**D**) and (**E**) (factors type and cell fraction) showed significant interaction (*p* < 0.0001), differences between rings (*p* < 0.0001) and between types of puncta (*p* < 0.0001). **F** Data from (**E**) displayed as cumulative histogram. A higher fraction of neutral LAMP1-positive puncta was found in the more central fraction of the cell (significant at fraction 60%)

Similar findings were obtained when we used the same paradigm on cells expressing TFL-tagged α-synuclein (Fig. 5A). As for LC3, we observed neutral “early” autolysosomes, which were positive for LAMP1 but with neutral pH as indicated by preserved green and red fluorescence (Fig. 5C). Again, these neutral/early autophagosomes were more abundant than acidic/mature autophagosomes and located preferentially in the cell center (Fig. 5E, F). In addition, we observed abundant acidic amphisomes in addition to neutral autophagosomes, characterized by retained red fluorescence, quenched green fluorescence, and negative staining for LAMP1 (Fig. 5B). Amphisomes were again located preferentially in the cell periphery (Fig. 5D). When we plotted for LAMP1-negative puncta (Fig. 5D) and for LAMP1-positive puncta (Fig. 5E, F) the location of neutral vs. acidic puncta, we observed a more peripheral location of the acidic puncta—as we did for LC3 (Fig. 4).

### Autophagosome-Lysosome Fusion Occurs in the Cell Center

**Fig. 6:**
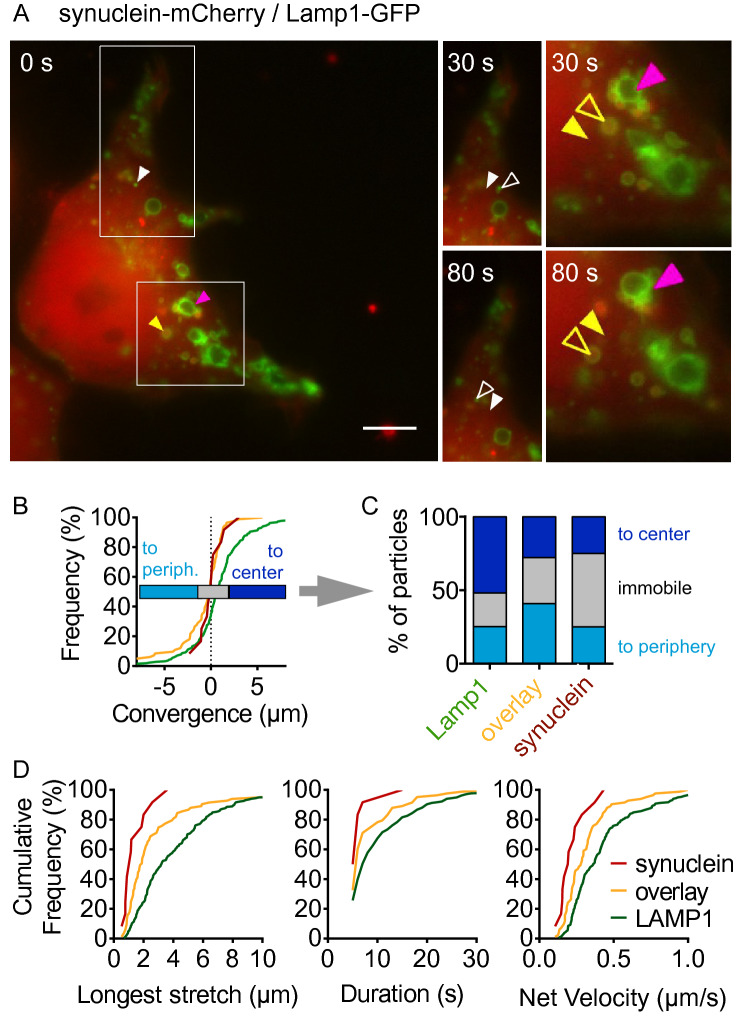
Transport of lysosomes and autolysosomes. **A** Sequential images of a living HEK293T cell expressing EGFP-tagged LAMP1 and mCherry-tagged A53T-α-synuclein at the beginning of recording (0 s). Insets show the indicated regions 30 and 80 s later. White arrowheads depict a small “LAMP1 only” particle (particles that appear green in the merged images), yellow arrowheads depict a large “overlay” particle (yellow in the merged images), and pink arrowheads depict a cluster. Filled arrowheads depict the position at the start of the recording, open arrowheads depict the position at later time points. At 30 s, the “LAMP1 only” particle has moved to the right of its original position. At 80 s, it has moved back and further to the left than its original position, illustrating the bidirectional movement observed for many particles. Similarly, the “overlay” particle has moved to the top right by 30 s, doubled back and moved further to the left than its original position. A movie of this cell is included as supplemental material. **B** Cumulative histogram of convergence values for non-clustered vesicles computed as in Fig. 2C. *n* = 139 α-synuclein puncta, representing free aggregates or autophagosomes, *n* = 405 LAMP1 puncta, representing lysosomes, and *n* = 236 overlay puncta, representing autolysosomes, from 64 cells and 4 independent experiments. **C** Data from (**B**) expressed as the percentages of puncta with movement towards the cell center (convergence > 0.5 µm), towards the cell periphery (convergence <  − 0.5 µm), or immobile (− 0.5 µm < convergence < 0.5 µm). Comparisons by *χ*^2^ test. *p* = 0.002 for “to center” vs “not to center” and lysosomes vs synuclein and autolysosomes. *p* < 0.0001 for “to periphery” vs “not to periphery” and autolysosomes vs lysosomes and synuclein. **D** Cumulative histogram of the longest stretch of directional transport observed for each particle during tracking (left), the duration of this transport (center), and the speed calculated as stretch/duration (right)

The location of early autolysosomes in the cell center suggests that this could be the location where fusion of autophagosomes with lysosomes occurs. In order to corroborate this hypothesis, we tracked fluorescent puncta in cells expressing GFP-tagged LAMP1 and mCherry-tagged A53T-α-synuclein (Fig. 6). Figure 6A shows a cell with a small LAMP1-positive particle (white arrowhead), a large “overlay” particle (yellow arrowhead), and a cluster of puncta (pink arrowhead). As in Fig. 2, filled arrowheads subsequently depict the position at the beginning of the recording and open arrowhead the position at later time points. Figures 6B and C summarize the results for many recordings of non-clustered puncta as defined above. All three types of vesicles moved in both directions, but the species moving towards the cell center most consistently were lysosomes (Fig. 6B, C). This observation is compatible with the hypothesis that lysosomes are made in the cell periphery (Yu et al. 2010; Li et al. 2016) and transported towards the cell center for fusion with autophagosomes. It is also consistent with the steady-state findings (Fig. 3B) where lysosomes were located further in the periphery than autolysosomes.

The species moving towards the cell periphery most consistently were autolysosomes (Fig. 6B, C), suggesting that autolysosomes are made in the cell center and transported towards the cell periphery, consistent with the finding that early autolysosomes are located more centrally than mature autolysosomes (Figs. 4G, 5F). The longest stretches of directional transport were observed for lysosomes, followed by autolysosomes (Fig. 6D). Lysosomes also moved for the longest duration, resulting in the fastest “net velocity”; α-synuclein particles moved least. The observation of long stretches of transport for the presumably acidic lysosomes is consistent with the long stretches observed for acidic LC3-tagged vesicles (Fig. 2G). The fact that transport differed between α-synuclein particles and autolysosomes (Fig. 6D) but not between neutral and acidic α-synuclein particles (Fig. 2F) supports again the notion that acidic vesicles and LAMP1-positive vesicles are overlapping populations of vesicles but not identical (Fig. 5).

### The Majority of α-Synuclein Puncta are Vesicles

**Fig. 7:**
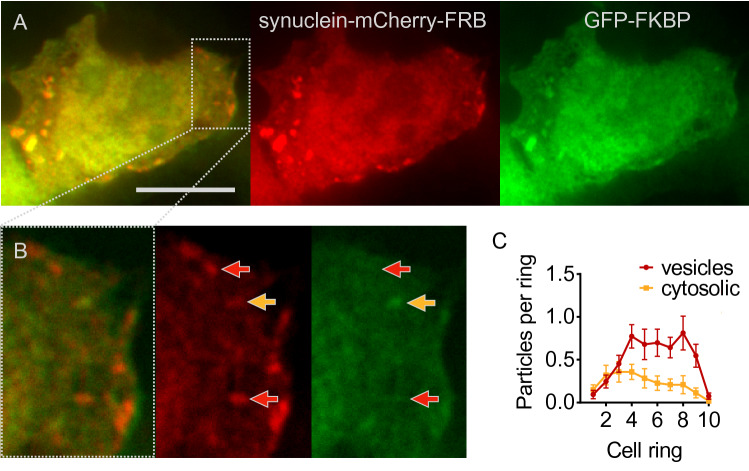
Discrimination between vesicles and cytosolic aggregates. **A** Image of HEK293T cells expressing A53T-α-synuclein (tagged by mCherry and FRB) and EGFP-FKBP. Cells were fixed 1 h after addition of 100 nM rapamycin. Depicted are merged images, red channel and green channel. Scale bar 10 µm. **B** Inset of area indicated in (**A**). The yellow arrow depicts the position of a cytosolic α-synuclein aggregate, i.e., a particle that is visible in both the red and the green channel, indicating that the mCherry-tagged α-synuclein colocalizes with EGFP-FKBP after rapamycin-induced dimerization of the FRB and FKBP domains. Red arrows depict the position of α-synuclein confined in vesicles, which is inaccessible for cytosolic EGFP-FKBP, causing puncta to be visible in the red channel but not in the green channel. **C** Average number of cytosolic aggregates (yellow) and vesicles (red) per cell ring from *n* = 53 cells as in (**A**) and 3 independent experiments. Two-way ANOVA (factors particle type and cell fraction) showed significant interaction (*p* = 0.0084), differences between rings (*p* < 0.0001) and between types of puncta (*p* < 0.0001)

As noted above, puncta observed with fluorescently tagged α-synuclein include both cytosolic aggregates and α-synuclein contained in autophagosomal vesicles. In order to discriminate between these two populations, we used chemically-induced dimerization as previously (Dinter et al. 2016): The small molecule rapamycin leads to rapid dimerization of two protein domains, FK506 binding protein (FKBP) and FKBP-rapamycin binding domain (FRB). When we expressed cytosolic FKBP-GFP with A53T-α-synuclein tagged by mCherry and FRB, the addition of rapamycin induced colocalization of GFP and mCherry at cytosolic α-synuclein aggregates (yellow arrow in Fig. 7B). In contrast, α-synuclein inside vesicles was inaccessible to GFP-tagged FKBP (red arrow in Fig. 7B). Based on this approach, we estimate that about 70% of fluorescent puncta are vesicles (Fig. 7C). The location of synuclein-labeled fluorescent puncta is thus dominated by vesicles and not aggregates, which explains the similarity between data obtained for LC3 and α-synuclein (Fig. 1D vs. E, and Fig. 4 vs. Fig. 5). Cytosolic α-synuclein aggregates were mainly found in the cell center and vesicles were found further in the cell periphery (Fig. 7C).

### Autophagosomes in Primary Astrocytes

**Fig. 8:**
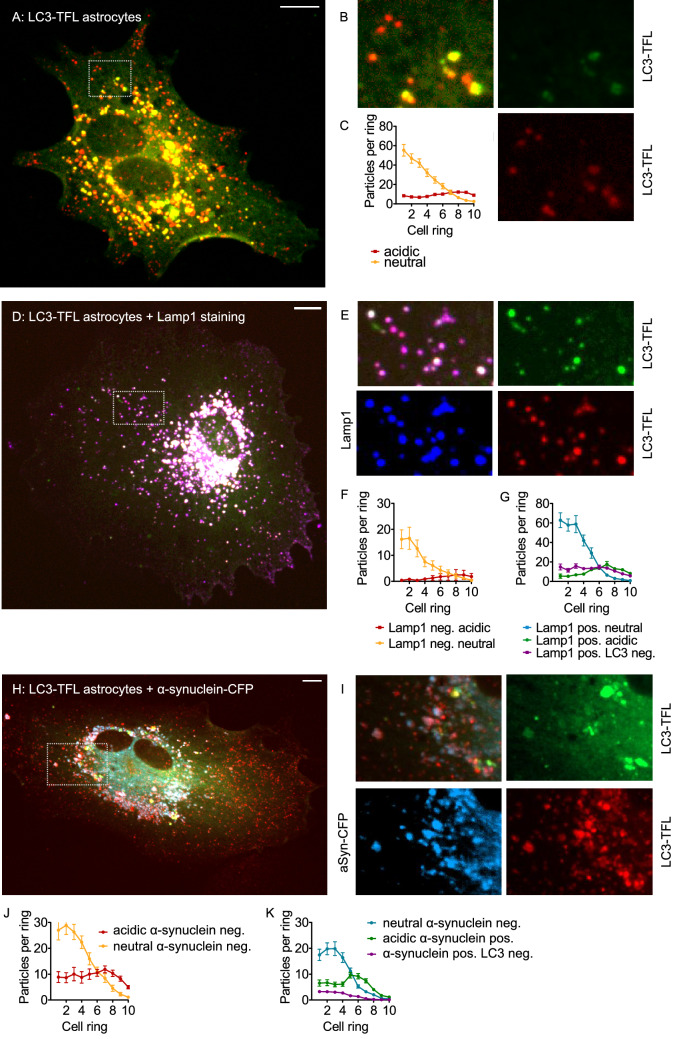
Autophagosomes in primary astrocytes. **A** Confocal image of fixed primary astrocyte expressing LC3-TFL, merged image of red channel and green channel. Scale bar 10 µm. **B** Enlargement of the area indicated in (**A**) showing in more detail the presence of “red” (upper left, negative in green channel) and “yellow” (lower right, positive in both channels) vesicles. **C** Average number of neutral autophagosomes (yellow) and acidic autophagosomes (red) per cell ring from *n* = 112 cells as in (**A**) and 3 independent experiments. Two-way ANOVA (factors particle type and cell fraction) showed significant interaction (*p* < 0.0001), differences between rings (*p* < 0.0001), and between types of puncta (*p* < 0.0001). **D** Confocal image of fixed primary astrocyte expressing LC3-TFL and stained for LAMP1, merged image of red channel, green channel, and blue channel (LAMP1). Scale bar 10 µm. **E** Enlargement of the area indicated in (**D**) showing in more detail the strong overlap between the three channels. **F** Average number of neutral (yellow) and acidic (red) puncta negative for LAMP1 per cell ring from *n* = 45 cells as in (**D**) and 3 independent experiments. Two-way ANOVA (factors particle type and cell fraction) showed significant interaction (*p* < 0.0001), differences between rings (*p* < 0.0001), and between types of puncta (*p* < 0.0001). **G** Average number of neutral (blue) and acidic (green) puncta positive for LAMP1 from the same cells as in (**F**). Lysosomes (LAMP1 positive, but no green or red fluorescence) depicted in purple. Two-way ANOVA (factors particle type and cell fraction) showed significant interaction (*p* < 0.0001), differences between rings (*p* < 0.0001) and between types of puncta (*p* < 0.0001). **H** Confocal image of fixed primary astrocyte expressing LC3-TFL and CFP-tagged α-synuclein, merged image of red channel, green channel, and cyan channel (α-synuclein). Scale bar 10 µm. **I** Enlargement of the area indicated in (H) showing in more detail the various particle types, including “cyan” puncta (α-synuclein only, cytosolic aggregates). **J** Average number of neutral (yellow) and acidic (red) puncta negative for α-synuclein per cell ring from *n* = 30 cells as in (**H**) and 3 independent experiments. Two-way ANOVA (factors particle type and cell fraction) showed significant interaction (*p* < 0.0001), differences between rings (*p* < 0.0001), and between types of puncta (*p* < 0.0001). **K** Average number of neutral (blue) and acidic (green) puncta positive for α-synuclein from the same cells as in (**J**). Cytosolic aggregates (α-synuclein-positive, but no green or red fluorescence) are depicted in purple. Two-way ANOVA (factors particle type and cell fraction) showed significant interaction (*p* < 0.0001), differences between rings (*p* < 0.0001) and between types of puncta (*p* < 0.0001)

In order to validate the findings obtained in HEK293T cells in a different cell type, we evaluated the subcellular distribution of neutral and acidic autophagosomes in primary astrocytes. Cultures were obtained from a mouse line expressing LC3-TFL from a CAG promoter. Primary astrocytes contained many more autophagosomes than HEK293T cells (Fig. 8A–C, compare to Fig. 1B, E). Yet, as in HEK293T cells, the cell periphery and in particular the “tips” contained mainly acidic autophagosomes, whereas the cell center and perinuclear region contained mainly autophagosomes with neutral pH (Fig. 8A, C).

In addition, we stained these astrocytes against the lysosomal marker LAMP1 (Fig. 8D–G). As in HEK293T cells (Fig. 4), LAMP1-negative neutral vesicles in astrocytes (yellow in Fig. 8F) were more commonly located in the cell center than Lamp1-negative acidic vesicles (red in Fig. 8F), which were mainly found in the cell periphery. The same difference was found for LAMP1-positive neutral vesicles (blue in Fig. 8G) and Lamp1-positive acidic vesicles (green in Fig. 8G). Yet, there were also differences between HEK293T cells and astrocytes: In HEK293T cells, LAMP1-positive autophagosomes were about as abundant as LAMP1-negative autophagosomes (Fig. 4E, F). In primary astrocytes, however, LAMP1-positive autophagosomes were more than seven times abundant than LAMP1-negative autophagosomes (note the different y-axis scale between Figs. 8F and G). Moreover, lysosomes (purple in Fig. 8G) were much less abundant in astrocytes than in HEK293T cells (Fig. 4F), i.e., more lysosomes are already fused to autophagosomes in astrocytes.

Finally, we compared autophagosomes with and without α-synuclein in primary astrocytes (Fig. 8H–K). To this end, astrocytes expressing LC3-TFL were transfected with CFP-tagged α-synuclein. These cells contained many different types of vesicles, including neutral and acidic autophagosomes, autophagosomes positive and negative for α-synuclein, and α-synuclein-positive puncta that were negative for LC3, most likely cytosolic aggregates (blue in Fig. 8I). For autophagosomes negative for α-synuclein, we found the familiar distribution of more central neutral vesicles (yellow in Fig. 8J) and more central acidic vesicles (red in Fig. 8J). Similarly, we also observed more central neutral vesicles (blue in Fig. 8K) and more peripheral acidic vesicles (green in Fig. 8K) for autophagosomes containing α-synuclein. Cytosolic α-synuclein aggregates (purple in Fig. 8K) were located primarily in the cell center, as we observed in HEK293T cells (Fig. 7C).

## Discussion

In this study, we determined subcellular location and mobility of vesicle species involved in aggregate clearance, focusing on two aspects of autophagosome maturation: fusion with lysosomes and vesicle acidification. Indeed, we observed systematic differences between autophagosomes, autolysosomes, and lysosomes, and between neutral and acidic autophagosomes. Collectively these findings suggest that both autophagosomes and autolysosomes form at the MTOC and move to the cell periphery during maturation.

### Specialized Cellular Regions for Aggregate Clearance by Autophagy

Our findings confirm—and expand—previous work demonstrating that individual steps of autophatic aggregate clearance happen in dedicated cellular regions. α-Synuclein aggregates accumulated in the cell center (graphical abstract). This is consistent with our previous observation that α-synuclein aggregates are transported towards the MTOC (Opazo et al. 2008), with the finding that autophagy of polyglutamine aggregates occurs in the cell center (Iwata et al. 2005), and with the concept that perinuclear aggresomes and JUNQ serve to concentrate cytosolic aggregates and orchestrate their degradation (Kopito 2000; Lamark and Johansen 2012; Hill et al. 2017). Starvation-induced autophagosomes, in contrast, form throughout the cell and subsequently move to the MTOC (Jahreiss et al. 2008; Korolchuk et al. 2011; Starling et al. 2016). The subcellular location where autophagosomes are formed thus differs between aggregate clearance and starvation-induced autophagy.

Three lines of evidence indicate that in our paradigm of α-synuclein aggregate clearance, fusion between autophagosomes and lysosomes mostly occurs at the MTOC, as observed for starvation-induced autophagy, and that autolysosomes are transported to the periphery during maturation. (i) We observed preferential transport of lysosomes to the cell center (Fig. 6C), consistent with the previous findings (Korolchuk et al. 2011; Starling et al. 2016) and with the fact that lysosomes are (re-)formed in the cell periphery (Yu et al. 2010; Li et al. 2016), where they were preferentially located (Fig. 3B). (ii) Neutral autolysosomes likely represent early or immature autolysosomes (see below). They were mainly found in the cell center (Fig. 4F, Fig. 5E, Fig. 8G), often near the aggresome (supplemental Figure S1), as were autolysosomes in general (Fig. 3B), consistent with the previous findings by others (Korolchuk et al. 2011). (iii) Autolysosomes—defined by colocalization of LAMP1 with α-synuclein—were preferentially transported to the cell periphery (Fig. 6C). (iv) This is consistent with the preferential transport of acidic autophagosomes and α-synuclein-containing vesicles towards the cell periphery (Fig. 2), and acidification is considered a core feature of autolysosomes.

The transport to the cell periphery of autolysosomes during maturation separates sites of lysosome reformation from sites of autolysosome fusion. A second purpose could be to secrete indigestible autophagosomal cargo, such as α-synuclein aggregates. Indeed, fusion of autolysosomal compartments with the plasma membrane was observed in different paradigms (Raiborg et al. 2015; Kimura et al. 2017; Matsumoto et al. 2018).

Two differences between autophagosomes labeled by LC3 and α-synuclein are worth noting. (i) The fraction of acidic puncta was larger for α-synuclein than for LC3 (Fig. 1D, E). The fraction of acidic puncta was also larger in primary astrocytes with α-synuclein (Fig. 8J) than without (Fig. 8C/F), but not much different between autophagosomes with and without α-synuclein of the same cell (Fig. 8J vs K). This finding can be explained by the recent finding that α-synuclein impairs degradation of acidic autophagosomes (Sarkar et al. 2021). (ii) LC3 vesicles were more mobile than α-synuclein puncta (Fig. 2C, E). This could reflect the inhibition of microtubule-dependent transport by α-synuclein (Prots et al. 2018). In addition, less mobile aggregates could contribute to the difference observed for neutral α-synuclein puncta.

### Reporting Vesicle pH by Tandem-Fluorescence

In our study, we used two methods to report autophagosome maturation, (i) colocalization with the lysosomal marker LAMP1 and (ii) luminal pH. For transport of mature autophagosomes/autolysosomes to the cell periphery, both approaches produced consistent results. A more detailed comparison, however, revealed some inconsistencies that prompted us to consider reporting of luminal pH in more detail.

α-Synuclein and LC3 were tagged with tandem-fluorescence (TFL), which exploits the difference in pKa between EGFP (pH 5.9) and mRFP (pH 4.5) (Zhou et al. 2014). This approach has been used widely since its original description (Pankiv et al. 2007; Kimura et al. 2007). Quantitative approaches to analyze the TFL signal have been described (Maulucci et al. 2015), but most groups just discriminate between “neutral” and “acidic” vesicles, as we did here. This straightforward analysis is an advantage of the TFL tag, but further studies could use ratiometric pH sensors like pHluorin2 (Mahon 2011) and correlate a continuous value for pH with distance from the MTOC or length of convergence movement. Caveats of the TFL tag include its large size of two fluorescent proteins. Moreover, the pKa of 5.9 for EGFP means that most vesicles with a pH of 6.5. will still be classified as ““neutral,” even though their pH is clearly different from 7.4. This fact can contribute to our finding of “early” autolysosomes that have already fused with LAMP1-containing lysosomes but still show “neutral” pH (Fig. 4C, 5C)—next to the notion that the internal membrane of autolysosomes initially remains intact after fusion with lysosomes (Tsuboyama et al. 2016). Autolysosomes classified as “neutral” by the TFL tag have also been reported by others (Tanida et al. 2014; Zhou et al. 2014). Why do these “early” autolysosomes appear more abundant than the classical “mature” autolysosomes (Fig. 4F, Fig. 5E)? When autolysosomes acidify to mature autolysosomes, the acidic pH activates lysosomal enzymes, which not only quench GFP but degrade the entire TFL tag. Mature autolysosomes are thus difficult to discriminate from lysosomes with this method. In addition, the process of lysosome reformation (Yu et al. 2010; Li et al. 2016) indicates that there is some continuity between mature autolysosomes and lysosomes.

### Luminal pH May Regulate Vesicle Transport Direction

All types of vesicles tracked in this work were found to move bidirectionally (Fig. 2, Figs. 6). This indicates that these vesicle types harbor in general molecular motors for both the directions of movement. Accordingly, the transport direction is often determined not by recruitment of motor proteins, but by their activation and inactivation (Monzon et al. 2020). In this context, it is tempting to speculate about the mechanisms that regulate the molecular motors and hence transport direction during autophagosome maturation. Lysosomes were preferentially transported to the cell center (Fig. 6C), as were neutral autophagosomes (Fig. 2E), both consistent with the literature (Jahreiss et al. 2008; Korolchuk et al. 2011; Starling et al. 2016). Hence, fusion of lysosomes with autophagosomes unlikely causes autolysosomes to move towards the cell periphery. We therefore propose that luminal pH itself could regulate the direction of vesicle transport.

Acidic autophagosomes (tagged by LC3) were located further in the cell periphery than neutral autophagosomes and moved to the cell periphery more often (Figs. 1, 2, 8), and this difference was even observed among either LAMP1-positive or LAMP1-neative autophagosomes (Figs. 4, 8). For α-synuclein, the difference between more central cytosolic aggregates and more peripheral vesicles (Fig. 7) could contribute to more peripheral location of acidic puncta as opposed to neutral puncta (Fig. 1), but the transport bias towards the periphery of acidic α-synuclein vesicles (Fig. 2B) and the different location of neutral and acidic vesicles containing α-synuclein and LAMP1 (Fig. 5E, F) are best explained by luminal pH. Luminal pH was indeed recently demonstrated to affect the lipid composition of phagosomes (Naufer et al. 2018), and lipid composition can regulate vesicle transport through motor protein clustering (Rai et al. 2016). Activation and deactivation of vesicular motor proteins by luminal pH therefore seems possible, but experiments in reduced systems will be required to convincingly demonstrate this mechanism.

## Supplementary Information

Below is the link to the electronic supplementary material.

Supplementary file1 (PDF 2660 kb)

Supplementary file2 (MOV 13629 kb)
