# Supplementary material for: Maturing Autophagosomes are Transported Towards the Cell Periphery
Source: Cell Mol Neurobiol. 2021 Jun 9;42(1):155–71. doi: 10.1007/s10571-021-01116-0 (PMC8732932; doi:10.1007/s10571-021-01116-0)
Supplement: Supplementary file 1 — Supplementary file1 (PDF 2660 kb) [file 10571_2021_1116_MOESM1_ESM.pdf]

<u>Supplemental Material to:</u>

Maturing autophagosomes are transported towards the cell periphery

Anna Hilverling[1], Elisabeth Dinter[2], Eva M. Szegö[2], Theodora Saridaki[1] and Björn H. Falkenburger[1,2,3,4]

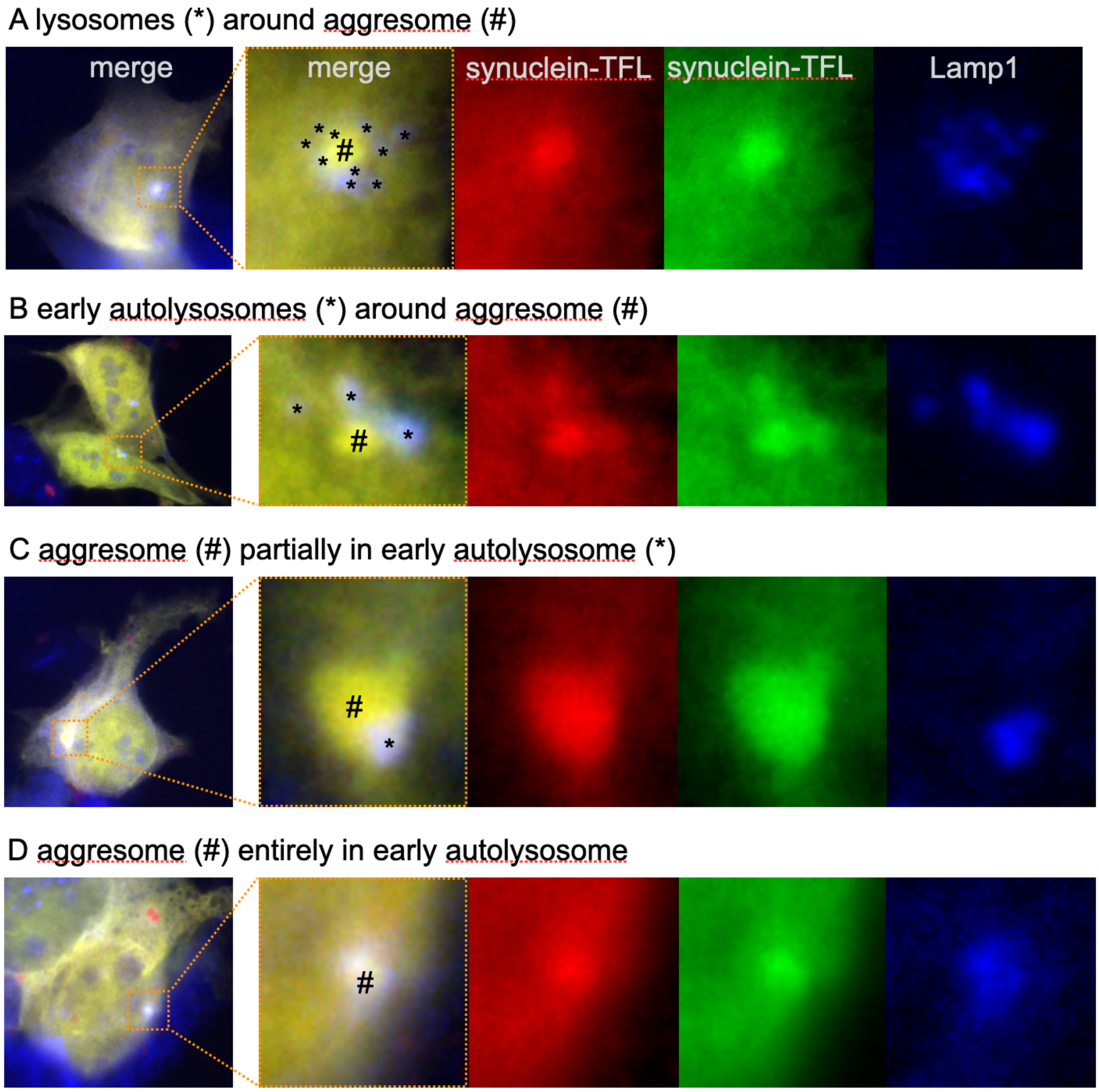

Supplemental Figure S1: Illustration of lysosomes and early autolysosomes located at the aggresome

Images of fixed HEK293T cells expressing TFL-tagged α-synuclein (red and green channels) and stained for Lamp1 (blue channel). The aggresome is a large, usually round accumulation of aggregates in the perinuclear region, at the MTOC. (**A**) Lysosomes are clustered around the aggresome, but the aggresome is Lamp1 negative. (**B**) The aggresome itself is Lamp1 negative, but two early autolysosomes, characterized by intact green fluorescence but positive for Lamp1, are located close to the aggresome. (**C**) Part of the aggresome is Lamp1-positive, suggesting that some of the aggregates clustered in the aggresome have been enclosed in autolysosomes. (**D**) Large perinuclear autolysosome, potentially corresponding to an engulfed aggresome. The observation of these early autolysosomes at the aggresome support the notion that both the engulfment of aggregates into autophagosomes and the subsequent fusion of autophagosomes with lysosomes occur at the MTOC.

Supplemental Text: ImageJ Macro to determine cumulative histograms for three types of puncta by using an eroding cell outline.

```
// This macro determines the subcellular positions and colocalisation of
// vesicles (or other markers) by repeatedly eroding an outline of the cell
// This variant works only with saved ROI sets, not with images
// before using it, save for each image the cell outline in the first
position
// of the ROI manager using the feehand tool, and save for each color
channel
// the localisation of vesicles using the multipoint tool

var imageX = 2464; // pixels of original image
var imageY = 2056; // pixels of original image
var increments = 20;  // percent increments during shrinking
var number = 100/increments;  // number of rings during shrinking
var hms = 2 // half marker size for drawing
inputDir    = getDirectory("Choose a Directory to load files ");
saveDir = inputDir;     // put results in same folder as original files
fileList    = getFileList(inputDir);
setBatchMode(true); // prevents showing images during macro

// cleaning up before starting
run("ROI Manager...");
roiManager("reset");
print("\\Clear"); // empty the log window
close("*");

//loop through all files of the folder inputDir
for (i = 0; i < lengthOf(fileList); i++) {
      fileName=fileList[i];

      if (endsWith(fileName, ".zip")&&!startsWith(fileName, "Res_")) {
            // if file is a ROI set and not a result file
            fileNameParts = split(fileName, ".");
            baseName = fileNameParts[0];
            print("Processing file: "+fileName);
            roiManager("Open", inputDir+fileName);
            channels = roiManager('count') -1;
```

```
            // create erosions
            newImage("erosions", "8-bit", imageX, imageY, 1);
            roiManager('select', 0);      // select cell outline
            getRawStatistics(area, mean, dummy, dummy, dummy, dummy2);
            original = area;  // size of original cell outline
            run("Create Mask");     // converts selction into b/w image
            mask_name = getTitle();
            for (j=1; (j<number); j++) {        // loop through rings
                  do {                    // erode by incements percent
                        run("Erode");
                        run("Create Selection");
                        getRawStatistics(area,m,d,d,d,d2);
                  } while (area>original*(1-increments*j/100));
                  roiManager("Add");// add outline to ROI manager
            }     // done with making erosions
            close("*");       // close all windows

            // do particle analysis
            Table.create("Puncta");
            Table.reset("Puncta");
            results = newArray(number);   // number of rings
            for (j = 1; j < 1+channels; j++) {  // loop through channels,
                                                // first is cell outline
                  // draw ovals from multipoint selection
                  newImage("PointCounting","8-bit white",imageX,imageY,1);
                  roiManager('select', j);
                  rName = Roi.getName();
                  print("processing channel: ", rName);
                  getSelectionCoordinates(x, y);
                  setColor(0); // black
                  l = x.length;
                  print("number of puncta in channel ", j, " : ", l);
                  for (k=0; k<l; k++)
                        fillOval(x[k]-hms, y[k]-hms, 2*hms, 2*hms);
                  // perform particle analysis for entire cell
                  roiManager('select', 0);      // first is cell outline
                  run("Analyze Puncta...", "display clear");
                  print("particle analysis entire cell : ", nResults);
                  results[0] = nResults;
                  // perform particle analysis for erosions
                  for (k = 1; k < number; k++) { // loop through erosions
                        roiManager('select', channels+k);
                        run("Analyze Puncta...", "display clear");
                        results[k] = nResults;
                  }
                  close("*");
                  Table.setColumn(rName, results, "Puncta");
            }     // end of loop through channels
            Table.save(saveDir + "Res_" + baseName + ".txt");
            roiManager("save", saveDir + "Res_" + fileName);
            roiManager("reset");
            print("  ");      // new line between files
      }     // end of: if file is ROI set
}           // end of loop through files
setBatchMode(false);
selectWindow("Log");  //select Log-window
saveAs("text", saveDir + "Log_ParticleAnalysis.txt");

// Notice of end of process
waitForUser("Process is done");
```

Supplemental Movie: Time-lapse microscopy of cell depicted in Figure 6A.
